# Extrathyroid carcinoma showing thymus-like differentiation (CASTLE): a new case report and review of the therapeutic role of neck dissection and radiotherapy

**DOI:** 10.1186/1477-7819-12-247

**Published:** 2014-08-03

**Authors:** Kyu Young Choi, Mi Jung Kwon, Hye Kyung Ahn, Jin Hwan Kim, Dong Jin Lee

**Affiliations:** 1 Department of Otorhinolaryngology-Head and Neck Surgery, Hallym University College of Medicine, Kangnam Sacred Heart Hospital, Daerim-1dong, Yeongdeungpo-gu, Seoul 150-950, South Korea; 2 Department of Pathology, Hallym University College of Medicine, Hallym University Sungsim Hospital, Pyeongan-dong, Dongan-gu, Anyang-si, Gyeonggi-do 431-796, South Korea; 3 Department of Pathology, Hallym University College of Medicine, Kangnam Sacred Heart Hospital, Daerim-1dong, Yeongdeungpo-gu, Seoul 150-950, South Korea

**Keywords:** Carcinoma showing thymus-like differentiation, CASTLE, thyroid gland, neck dissection, radiotherapy

## Abstract

We present here a case of extrathyroid CASTLE (the third case reported in the English literature) treated with excision and neck dissection without radiotherapy. Also, we reviewed the literature and analyzed the therapeutic results of each treatment modality for CASTLE. A 27-year-old male had initially presented with a painless, right neck mass for 2 months. Computed tomography of the neck showed a 3.8 × 3.2 × 3.8 cm heterogeneously enhancing mass at right level IIa, and no definite thyroid lesion was found. An excisional biopsy was done and the pathologic diagnosis was CASTLE. Then we performed a right modified radical neck dissection and right thyroid lobectomy. After three years, no evidence of tumor recurrence was noted. Total excision followed by neck dissection could be a sufficient surgical treatment option for CASTLE. Postoperative radiotherapy might be an alternative treatment option for neck dissection in patients with positive nodal status.

## Background

Carcinoma showing thymus-like differentiation (CASTLE) is a rare tumor of the thyroid gland or adjacent soft tissues of the neck [1]. First described as an intrathyroid epithelial thymoma by Miyauchi *et al.* in 1985 [2], these lesions were later designated CASTLE by Chan and Rosai, who proposed an origin from ectopic thymus, or from thymopharyngeal duct or branchial pouch remnants in the thyroid gland and neck [3]. The majority of tumors occur in the thyroid gland, usually in the lower portion, and they rarely arise in the extrathyroidal soft tissue of the neck. The histopathology of CASTLE is characterized by an expansive growth pattern, thick fibrous bands dividing the tumor nests, the presence of lymphocytes and rare or infrequent mitoses [4]. Generally, the treatment is surgical excision with or without radiotherapy, but the definitive treatment remains uncertain, due to the rarity of the disease.

According to reports in the English-language literature, less than 30 cases of CASTLE tumors have been reported, and only two cases were extrathyroid tumors [5]. One case was reported by Ahuja in 1998, in which a female patient with a lateral neck CASTLE was treated by resection and radiotherapy without recurrence [6], and the other case was described by Luo, in which a lateral neck CASTLE was treated without locoregional recurrence by surgical excision and radiotherapy [5].

Here, we report a case of extrathyroid CASTLE, which was diagnosed by excisional biopsy, successfully treated by unilateral neck dissection with ipsilateral thyroid lobectomy, without concomitant radiotherapy.

## Case presentation

**Figure 1:**
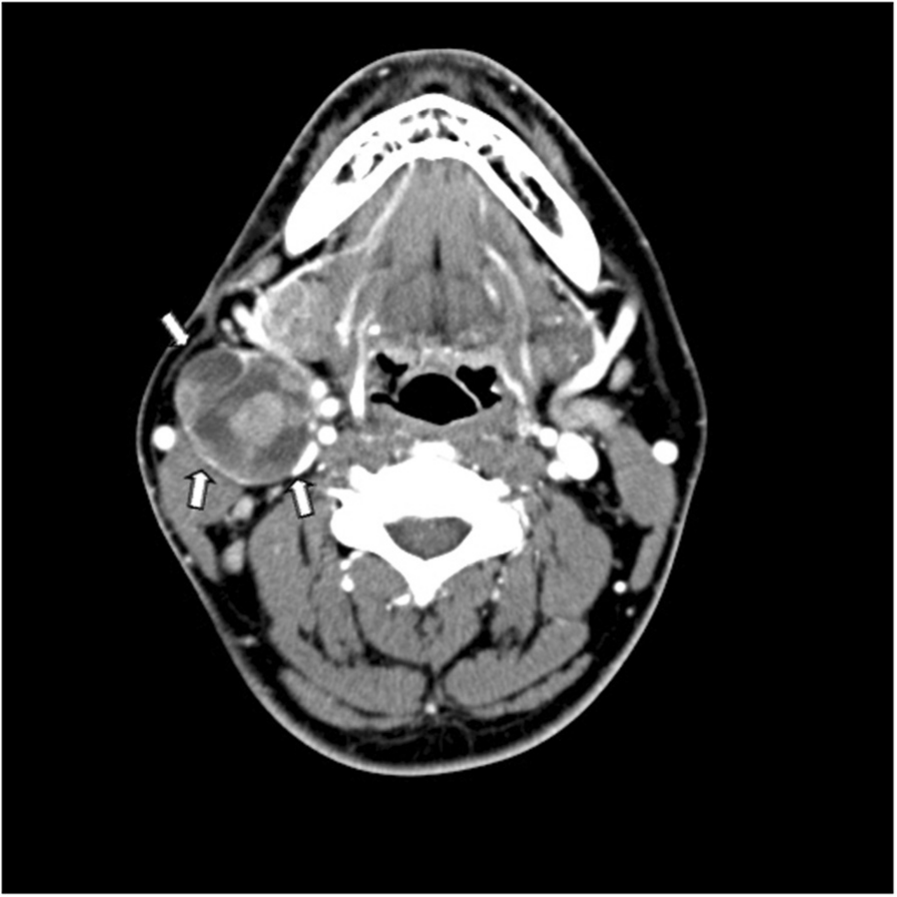
**Computed tomography (CT) findings.** CT with enhancement showing heterogeneously enhancing mass at right level II (arrows, tumor margin).

**Figure 2:**
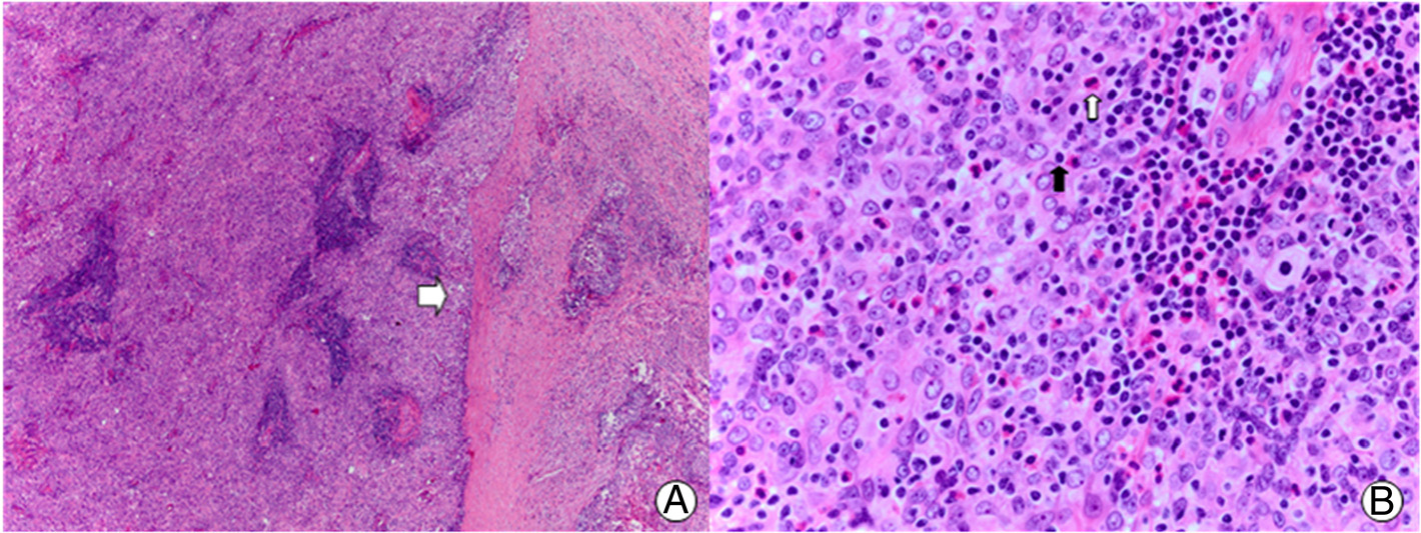
**Histopathological features of the tumor. (A)** Tumor cell nests separated by fibrous bands (white arrow, hematoxylin and eosin stain; original magnification × 40), **(B)** with accompanying lymphocyte (black arrow) and eosinophil (white arrow) infiltration (hematoxylin and eosin stain; original magnification × 400).

A 27-year-old male was admitted to our department with a palpable neck mass that appeared two months ago. He denied any other symptoms. His past medical history was significant only for left chronic otitis media with mild hearing disturbance, and both chronic sinusitis, which was treated surgically by endoscopic sinus surgery seven years ago. On physical examination, the 3-cm-sized mass was hard and fixed, locating at right level IIa. No obvious lesion was found in the oral cavity, nasopharynx, oropharynx or hypopharynx. Laboratory tests showed a white blood cell count of 7,480 mm^3^ with 64.2% neutrophils, and the serum concentrations of thyroxine, thyroid-stimulating hormone and thyroglobulin were all normal. Neck ultrasonography revealed multiple tiny cystic-hypoechoic nodules in both thyroid glands, and a 3.8 × 3.2 × 3.8 cm mass with heterogeneous echogenicity in the right submandibular area. Computed tomography (CT) with enhancement of the neck showed the same-sized heterogeneously enhancing mass at right level IIa (Figure 1). Ultrasonogram guided fine-needle aspiration (FNA) biopsy of the mass was performed and only atypical plump spindle cells were found. We performed a second FNA biopsy after two weeks. The cytologic examination showed plump epitheloid and spindle cells with infiltration of lymphoid cells and eosinophils. An immunohistochemical stain of the cytology showed that the tumor was positive for keratin, high-molecular keratin, vimentin and galectin-3, and focally positive for Factor VIII.To make a definite diagnosis, we scheduled an excisional biopsy of the mass. Under general anesthesia, a skin incision over the mass and subsequent dissection of the platysma muscle was done, and the tumor appeared just inferior to the right submandibular gland. The tumor was well encapsulated with several lymph nodes around it. Excision of the mass along with six right level IIa lymph nodes was performed. No other specific intraoperative findings were noticed and the frozen biopsy revealed not much and no malignant tissue was found. One week after, a final report of the permanent pathology was made and the diagnosis was carcinoma showing a thymus-like element. Histological examination of the mass showed an infiltrative neoplasm composed of epithelial cell nests separated by thick fibrous septa, with an accompanying infiltrate of small lymphocytes and eosinophils (Figure 2). Immunohistochemical stains of the mass for CD5, cytokeratin and P63 were positive, and negative for thyroid transcription factor-1. The six lymph nodes had no tumor present.

The patient refused radiotherapy, but agreed on complete surgical eradication of the disease. Because of the patient’s young age, we scheduled a right modified radical neck dissection, along with ipsilateral thyroid lobectomy to rule out a thyroid origin. Preoperative positron emission tomography-computed tomography (PET-CT) showed no metabolic evidence of malignancy, and an ultrasonogram guided thyroid nodule FNA biopsy result was non diagnostic. Finally, two months after the first operation, a right modified radical neck dissection (levels I to V) and ipsilateral thyroid lobectomy were performed. A thyroid lobectomy was added to our surgery because most CASTLE tumors are intrathyroid, and also to give a definite diagnosis of the patient’s thyroid nodule. On intraoperative inspection and palpation of the thyroid, no noticeable intrathyroid mass was found. The surgery was finished without difficulty, and no other specific intraoperative findings were noted. The pathologic report of the right thyroid lobe was multinodular goiter and no definite neoplastic lesion was identified. All the lymph nodes in right levels I to V were non-neoplastic (reactive hyperplasia). The patient was discharged on the ninth hospital day without any postoperative complications, and was then regularly followed up in the outpatient department. After six months, a follow up PET-CT was taken and no metabolic evidence of regional tumor recurrence or metastasis was reported. At the most recent follow-up, i.e. three years after the second operation, no evidence of tumor recurrence was present.

## Discussion

CASTLE is a rare malignant neoplasm that histologically resembles thymic carcinoma, and arises in the thyroid gland or adjacent soft tissue of the neck. During embryological development, primordial thymic tissue derived from the third and fourth branchial pouches migrates through the neck and into the mediastinum [7]. Persistence of thymic remnants along this path can manifest as ectopic thymic tissue [3]. CASTLE is postulated to arise from either this ectopic tissue, from remnants of the thymopharyngeal duct or from ultimobranchial body remnants within the thyroid gland [3,8]. According to previous reports, most CASTLE tumors are located in the thyroid gland, especially the lower portion. From a review of literature, only three CASTLE tumors have been reported in the lateral neck including this case [5,6]. Table 1 summarizes the clinical characteristics, treatments and clinical outcomes of the three patients with extrathyroidal CASTLE.

**Table 1:** Clinical characteristics, treatments, and outcomes of patients with extrathyroidal origin CASTLE

**Cases**	**Ahuja *et al. *** **[**[6]**]**	**Luo *et al. *** **[**[5]**]**	**This case**
Age (years)/sex	67/female	47/male	27/male
Tumor size	9 × 7 × 5 cm	5 × 5 × 4 cm	3.8 × 3.8 × 3.2 cm
Location in neck	Left	Left	Right
Cystic changes	Not described	Partial	Partial
Inflammatory cell infiltration	Numerous lymphocytes	Lymphocytes	Abundant lymphocytes and eosinophils
Adjacent LNs	Multiple enlarged LNs adjacent to the mass	Several small LNs surrounding the mass	Multiple enlarged LNs adjacent to the mass
LN pathology	LN metastasis (1/22)	No tumor	No tumor
Treatment	Mass excision, LN excision, radiotherapy	Mass excision, LN excision, radiotherapy	Mass excision, right modified radical neck dissection (levels I–V)
Outcome	No recurrence	No recurrence	No recurrence over 3-year period

LN, lymph node.

An FNA biopsy seems to offer limited diagnostic value for CASTLE. Youens *et al.* identified 27 cases of reported CASTLE that mentioned an FNA biopsy [1]. Reported cytological differential diagnoses vary, with the majority of cases being identified as a malignant tumor, without further definite classification. One reported case was identified as suggestive of spindle cell neoplasm, one was identified as a papillary carcinoma, and one was correctly identified as CASTLE preoperatively. In 23 of 27 reported cases, CASTLE was correctly identified as malignant, and in one case, the FNA was negative. In our case, the FNA biopsy showed epitheloid and spindle cells with infiltration of lymphoid cells and eosinophils. CASTLE can be definitively diagnosed only by experienced pathologists through histologic examination. CASTLE produces a lobulated and expansive growth pattern with fibrous septa dividing the tumor, large vesicular nuclei with prominent nucleoli, a low mitotic count, and may be associated with lymphoplasmacytic infiltration [2,3,9], as seen in our patient (Figure 2). Small focal squamous differentiation in whorled clusters, resembling Hassall’s corpuscles, may also be seen [5].

In their study, Luo *et al.* found 26 reported cases of CASTLE, 25 intrathyroid cases, one extrathyroid case, and added one extrathyroid CASTLE, which they reported [5]. The 25 cases of intrathyroid CASTLE were treated with surgical excision (thyroidectomy, neck dissection, etc*.*) with or without radiotherapy. A review of all reported cases showed that 16 of 27 patients had no evidence of recurrence at follow-up after treatment, and 10 of 27 had a recurrence. There was one case of recurrence with initial misdiagnosis. Four of the ten recurrent cases showed no evidence of recurrence after salvage treatment; one patient died of a cerebrovascular disorder, and five died of the disease with local recurrence or distant metastasis to the lung or liver. Most of the recurrences were noted in patients with invasion of the neck lymph nodes or surrounding tissue. Two cases of extrathyroid CASTLE were treated by resection and radiotherapy, and neither showed locoregional recurrence.

There is no consensus on the management of CASTLE, due to the rarity of the disease, but complete surgical excision with or without radiotherapy seems necessary to improve the long-term survival rate and reduce the locoregional recurrence rate. Table 2 summarizes the treatment modalities and therapeutic results of CASTLE, from a review of the literature. We added six new cases from the recent literature for which we could find the treatment modalities and therapeutic results [10-13]. In cases with a tumor excision only, tumor recurrence occurred in six cases and there was no evidence of disease (NED) for seven cases. But for cases who had either radiation therapy or neck dissection after complete excision, recurrence occurred only for three cases and there was NED for nine cases. On the basis of these case reviews, we noticed that excision with either radiation or neck dissection showed better results than excision only. Additionally, if there is a positive node in the neck dissection specimen, postoperative radiation might be helpful as this adjuvant therapy reduced the recurrence rate from 100% to 57% (1/1 to 4/7). In contrast, if there is no metastatic node in the neck dissection specimen, postoperative radiation therapy does not seem necessary as recurrence has never occurred in node negative cases after neck dissection.

**Table 2:** **Treatment modalities and therapeutic results of reported CASTLEs**[5,6,10-13]

**Treatment modality**		**Recurrence**		**NED**
Excision only		6		7
Excision + RT		2		4
Excision + RT*		0		1
Excision + ND	N (+)	1	N (+)	0
	N (-)	0	N (-)	3
Excision + ND*	N (-)	0	N (-)	1
Excision + ND + RT	N (+)	4	N (+)	2
	N (-)	0	N (-)	2
Excision + ND + RT*	N (+)	0	N (+)	1
Total		13		21

*The three cases of extra-thyroid CASTLE.

N (*+*), node positive in neck dissection specimen; N (-), node negative in neck dissection specimen; ND, neck dissection; NED, no evidence of disease; RT, radiation therapy.

In summary, the most successful treatment from a review of the literature was complete surgical excision followed by neck dissection with or without postoperative adjuvant radiation therapy depending on nodal state. In our case, complete excision by neck dissection without radiotherapy achieved a positive result as there was no metastatic node in the neck specimen. Interestingly, all extrathyroid CASTLE cases (‘^*^’ in Table 2) showed NED. All three cases were treated by excision with either radiotherapy or neck dissection. The reason for the better prognosis compared with intrathyroid CASTLE might be the isolated origin site of extrathyroid CASTLE, and the higher feasibility of complete excision than intrathyroid CASTLE, which extends to the mediastinum. Extrathyroid CASTLE is difficult to diagnose, and there is no reported suitable treatment. In this case, the patient was diagnosed by excisional biopsy, and a subsequent unilateral neck dissection with ipsilateral thyroid lobectomy achieved a disease-free state. However, a longer follow-up in a larger series of patients is needed to support our result.

## Conclusions

We present a case of extrathyroid CASTLE, which was successfully treated by excision plus unilateral neck dissection. From a review of the literature, the most successful treatment of CASTLE is complete surgical excision followed by neck dissection with or without postoperative adjuvant radiation therapy depending on nodal state.

## Consent

Written informed consent was obtained from the patient for publication of this case and for the accompanying images.

## Abbreviations

CASTLE: carcinoma showing thymus-like differentiation; CT: computed tomography; FNA: fine-needle aspiration; NED: no evidence of disease; PET-CT: positron emission tomography-computed tomography.

## Competing interests

None of the authors involved in manuscript preparation have any competing interests regarding the manuscript itself, nor are there any financial or moral conflicts. None of the authors received support in the form of grants, equipment or pharmaceutical items.

## Authors’ contributions

KYC collected data and drafted the manuscript. MJK and HKA supervised the pathology and managed the figures. JHK supervised the writing of the manuscript. DJL conceived of the study and corrected and revised the manuscript. All authors read and approved the final manuscript.
